# The influence of climate change on growth of Arctic charr (*Salvelinus alpinus*)

**DOI:** 10.1007/s10641-025-01743-2

**Published:** 2025-09-08

**Authors:** Haley K. Gendron, Ross F. Tallman, Margaret F. Docker

**Affiliations:** 1 Department of Biological Sciences, University of Manitoba, Winnipeg, Canada; 2 Arctic and Aquatic Research Division, Fisheries and Oceans Canada, Winnipeg, Canada

**Keywords:** *Salvelinus*, Climate Change, Growth, Arctic, Otolith, Arctic charr

## Abstract

The Arctic is warming at an unprecedented rate and with longer growing seasons, greater rainfall, and less snowfall. Cold-adapted ectotherms, such as the Arctic charr, *Salvelinus alpinus* (Linnaeus 1758), are likely to experience changes to growth as a result. Anadromous Arctic charr (charr, hereafter) are of great importance for northern communities, providing a source of income from commercial fisheries and food security from subsistence harvest. Initially, warming is expected to increase the growth of charr, benefitting subsistence and commercial fisheries in the short term. However, over longer time scales, temperatures exceeding the optimum for growth will likely result in metabolic stress, slowed growth, and higher mortality. Thus, the long-term consequences of climate change will likely be negative. We assessed anadromous charr growth from 1984 to 2013 in three stocks around Cumberland Sound using otolith measurements as proxies for age-specific growth. Trend analyses indicated growth had increased in pre-migratory ages over the years. We used mixed models to investigate changes to growth for ages 1–10 in relation to climate variables, finding that growing degree days had the greatest positive influence on ages 1–6 while annual precipitation had an overall negative effect on growth in ages 1–2 and 6–10. Contrary to previous assessments on these stocks, our results suggest charr have indeed experienced changes to growth with climate change. These findings emphasize the need for more thorough long-term growth studies in the management of fisheries as altered growth will affect food security and the economy across the Canadian Arctic.

## Introduction

Arctic charr, *Salvelinus alpinus* (Linnaeus, 1758), charr hereafter, is a highly diverse species with a circumpolar distribution (Weinstein et al. 2024). It is a slow-growing and relatively long-lived species, due to its high latitude range, where average water temperatures are low and the growing season is short (Grainger 1953; Johnson 1980). Resident and anadromous ecotypes co-exist in freshwater environments; however, anadromous individuals migrate to sea during the summer months to feed, during which time much of their growth occurs (Grainger 1953). Anadromous charr then return to freshwater in the fall to spawn and overwinter (Grainger 1953). Overwintering typically occurs in freshwater systems due to poor tolerance of the sub-zero marine temperatures where they are thought to remain fairly dormant to conserve energy (Wandsvik and Jobling 1982; Finstad et al. 1989; Mulder et al. 2018a). Anadromous charr reaches a greater maximum length than their resident counterparts (Tallman et al. 1996; Loewen et al. 2010; Young et al. 2021), likely resulting from increased food availability at sea (Näslund 1990). As a result, anadromous charr are of commercial and subsistence importance across the Canadian Arctic (Babaluk et al. 2010; Roux et al. 2011). These fisheries are an essential ecosystem service in terms of economy, culture, and food security for Inuit and northern communities across Canada (Kuhnleini and Soueida 1992; Myers et al. 2005; Kenny et al. 2018; Falardeau et al. 2022). Commercial charr fisheries in Nunavut are relatively small-scale but are considered reliable with a market value of $1.8 million (CAD) in 2015 (Roux et al. 2011; Department of Environment, Fisheries and Sealing Division 2017; Castaneda et al. 2020). Charr stocks in northern Canada are co-managed by Fisheries and Oceans Canada (DFO) and communities via Inuit land management boards, integrating traditional ecological knowledge and western science in the management of both commercial and subsistence fisheries (Tallman et al. 2019).

Annual precipitation and air temperature have increased in the Arctic (AMAP 2021), and both have been found to affect charr growth (Hesthagen et al. 2004; Kristensen et al. 2006; Murdoch et al. 2015). The most notable of the two factors is temperature, whether it be of air or water, as the Arctic continues to warm at an accelerated rate (AMAP 2021). Much of the research on climate and growth in charr focuses on identifying thermal limits and optimum temperatures for growth (e.g., Lyytikäinen et al. 2002; Beuvard et al. 2022; Gilbert et al. 2022). An upper thermal limit of 21 °C is consistently documented in the literature and characterized by reduced growth, increased mortality and arrhythmia (Larsson and Berglund 2005; Gilbert et al. 2020; Beuvard et al. 2022). However, optimum temperatures for growth are not always consistent across the literature, possibly due to differing methods (i.e., laboratory vs. field-based) or varying thermal tolerance between populations. The optimum temperature for growth has been reported anywhere from 10.3 to 16.3 °C (Lyytikäinen et al. 2002; Larsson and Berglund 2005; Beuvard et al. 2022). These studies suggest that the growth of charr may increase with rising temperatures, until an optimum temperature is reached, after which point the growth rate is expected to decline. Changes to individual growth will impact both commercial and subsistence harvest, as quotas in northern Canada are based on the estimated biomass of the stock. Consequently, larger charr may increase the available biomass of the stock, allowing for larger harvests. Despite a handful of experimental studies, there are limited studies investigating the relationships between climate and growth in natural settings. This is an important factor, as wild charr live in complex, heterogenous systems and can behaviourally thermoregulate by moving to different thermal habitats. In addition, the application of these thermal limits and optimums for inferring climate-driven changes to growth is difficult without long-term water temperature datasets. Air temperature records, on the other hand, are widely available for much of the planet, including remote regions of the Arctic. Based on the climate changes already seen in the Arctic, it is possible charr have already been experiencing impacts. If not, it is probable in the future as climate change continues.

A commonly used method for estimating age-specific annual growth of fish is measuring annual growth bands, or annuli, of otoliths. This method is based upon the assumption of a proportionate relationship between fork length and otolith length in charr (Svenning et al. 1992). Otoliths are calcified structures found in the inner ear of bony fishes, aiding in balance and orientation (Pannella 1971; Campana 1999). One annulus is comprised of a translucent winter band and an opaque summer band, together representing 1 year of growth. As such, measurements from the nucleus to the end of each annulus can be used as a proxy for length-at-age and the measurements of individual annuli a proxy for annual growth. The archived otoliths used in this study were originally collected by DFO to analyze age structures and growth trends for stock assessments (e.g., DFO 2005, 2013, 2023). Thus, insights from this study will be relevant for management practices as changes to individual growth rate will have direct implications for both commercial and subsistence quotas.

The objective of this study is to investigate the growth of Cumberland Sound anadromous charr relative to climate change between 1984 and 2013 by (1) determining which climatic factors influence growth during this period, and (2) determining the nature of variation in growth between stocks and age classes. We predicted that (1) temperature and precipitation would have significant positive influences on growth, and (2) growth has increased over time in all three stocks with differences in growth between stocks attributed to early life habitat variation.

## Methods

### Study area

**Fig. 1:**
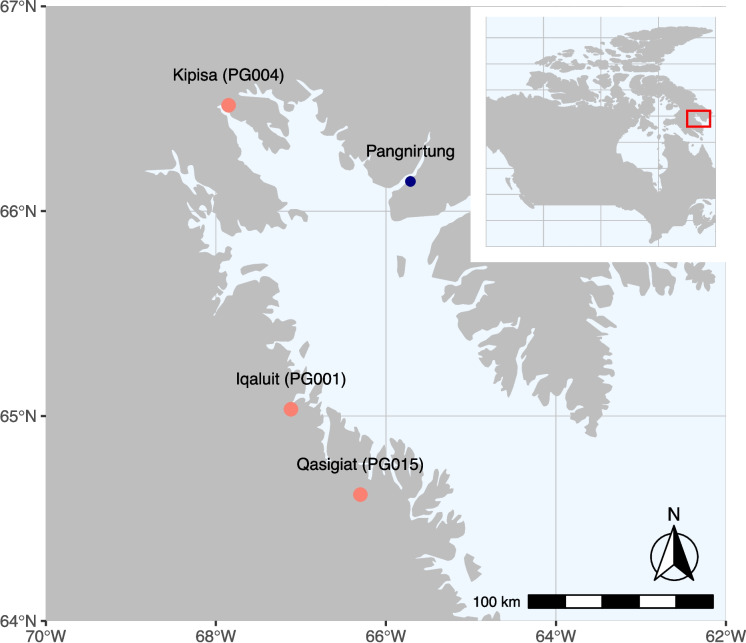
Map of Cumberland Sound, showing the nearest community, Pangnirtung, and the three study stocks: Kipisa (PG004), Iqaluit (PG001), and Qasigiat (PG015). The inset map shows the location of Cumberland Sound within Canada

Cumberland Sound is located on the southeastern coast of Baffin Island, Nunavut. The Sound is 300 km long and averages 65 km wide (Roux et al. 2019; Tallman and Marcoux 2021). Its water originates from both Arctic and North Atlantic water masses (McMeans et al. 2012). Depth ranges from 200 to 1000 m (Tallman and Marcoux 2021). The steep sides of the Sound (> 2125 m) are lined with deep fiords, connecting the marine environment to freshwater river and lake systems (Roux et al. 2019; Tallman and Marcoux 2021). Three of these lakes, corresponding to discrete stocks, located along the south side of Cumberland Sound, were selected for this study: Kipisa (PG004), Iqaluit (PG001), and Qasigiat (PG015; Fig. 1). The stocks in this region are relied on both for subsistence and commercial harvest by the hamlet of Pangnirtung. DFO waterbody codes are included in parentheses for consistency across the literature, since common lake names vary in the region. Qasigiat Lake (64°62′N, 66°31′W) is unusual in that it has a very short river connecting the marine environment to the lake. At high tide, there is virtually no separation between the two environments, leading to an inflow of saltwater into the lake (T. Loewen, personal communication, 29 November 2023). Iqaluit Lake (65°2’N, 67°7’W) is a large deep lake, connected to the marine environment via a 1.3-km long river (Martin et al. 2023). Kipisa Lake (66°52’N, 67°85’W) is the largest of the three lakes in terms of surface area and has the longest river (approximately 6.4 km) connecting the lake to the marine environment.

### Data collection

#### Biological and otolith growth data

Sagittal otoliths were used to examine annual growth over individual lifespans. Archived otoliths from 594 individuals were selected from the three stocks in two different sample years approximately 10 years apart to create a time series of growth radii and annuli (Kipisa – 2002 & 2011; Iqaluit – 2001 & 2013; Qasigiat – 2003 & 2011). These otoliths were collected as part of stock assessment initiatives (e.g., DFO 2005, 2013, 2023). Sampling for stock assessments is conducted as they are requested and rarely coincide with assessments on other stocks. As such, we were unable to use the same sample years for each stock due to data availability. However, this does not introduce significant issues as the back-calculated data represents a larger time series with considerable overlap between sample years and stocks.

All fish were caught in gill nets of various mesh sizes. For Iqaluit 2001 and Kipisa 2002, mesh sizes of 139.7 mm were used. In Qasigiat 2003, a combination of 139.7-mm mesh and multi-mesh (38–120 mm) nets were used. In 2011, Qasigiat was sampled with 38.1-mm mesh and multi mesh nets. The remainder of the sampling (Kipisa 2011 and Iqaluit 2013) was done using multi-mesh nets. Sampling occurred in freshwater at Iqaluit Lake in 2013 and Qasigiat in 2003 and 2011, when anadromous charr had returned to the lake for spawning and overwintering. Marine sampling took place at Iqaluit Lake in 2001 and Kipisa in 2002 and 2011, during marine residency. Fish were dissected on-site to record sex, maturity, fork length, life history, and weight. At this time, sagittal otoliths were collected for age estimation. Only those that were determined to be anadromous were included in this study.

When left and right otoliths were available, both were examined in a shallow dish of water under a Leica M125 microscope at 10 × magnification for initial screening of otolith quality. Otoliths were rejected when crystallization was observed. The otoliths in the best condition or with a flat or wide peak on the dorsal lobe were selected for embedding. Flat or wide peaks on the dorsal lobe are a prime location for sectioning as there is a lower margin of error in the placement of the saw blade, resulting in better sections with easy-to-distinguish annuli. Debris was cleaned from the otolith using precision tweezers under the microscope and a lateral grind was performed to improve clarity of the nucleus and first annulus. A two-part epoxy was mixed and poured onto a labelled strip of Parafilm® before the otolith was placed into the epoxy, sulcus side up. The epoxy was then left to cure in a fume hood for at least two days before sectioning. Once cured, the sectioning plane was marked under a microscope with a micrometre eyepiece using an Ultra Fine Tip Sharpie®. A true transverse section was generally used, although a slight anterior or posterior rotation was also used in some cases to achieve a better section. Sectioning was accomplished using a Buehler IsoMet™ Low-Speed Saw with a single saddle chuck set at a speed of 10. Two diamond wafering blades separated by a thin plastic spacer were used to cut a thin section from the embedded otolith.

Sections were photographed using a Leica M125 microscope fitted with a Leica Flexacam C3, with a magnification that allowed the ventral lobe and nucleus to fill most of the frame. A 1-mm scale bar was added at the time of image capture to account for different levels of magnification. The RFishBC package (v0.2.4; Ogle, 2022) was used to measure the distance between each annulus (annulus lengths; mm). These annulus lengths are used as a proxy for annual growth. Measurements were taken along a transect close to the ventral edge of the sulcus, extending from the nucleus to the edge of the otolith. This region was selected for measurements due to the consistent spacing and clarity of annuli. We were not able to identify the margin between annulus 1 and the nucleus with certainty, meaning the first year of growth could not be isolated from larval growth. As such, age-1 radius length (mm) will be used to assess growth in the first year (Svenning et al. 2024).

#### Climate and geographic data

Overall, the Canadian Arctic is a data-limited region due to the high costs associated with Arctic travel and poor weather conditions, making it difficult to obtain long-term water temperature datasets. However, studies have found that climate warming directly results in lake warming, and data derived from air temperatures can be used as a surrogate for water temperature in freshwater systems (Mooij et al. 2008; Kirillin 2010; Johnson et al. 2014). As such, growing degree days (GDD) derived from air temperature, are used as a surrogate for water temperature data.

Growing degree days (GDD; °C·Day) is a popular metric for describing growth in relation to temperature in ectotherms, such as fish (Neuheimer and Taggart 2007). The Climate Atlas of Canada (2019) describes growing degree days as the “annual sum of the number of degrees Celsius that each day’s mean temperature is above a specified threshold temperature” (Eq. 1). Thus, GDD effectively combines time and temperature into a single metric, which has been shown to better describe growth in fish than temperature alone (Neuheimer and Taggart 2007; Honsey et al. 2019). Growth in charr slows drastically or even stops during winter months while overwintering in fresh water (Mulder et al. 2018a). Water temperatures during this time are typically 0.2–2 °C (Klemetsen et al. 2003; Mulder et al. 2018b). Because maximum heart rate drops rapidly below 4 °C, likely indicative of a slowing metabolism like that seen during overwintering, a standard base temperature of 4 °C was selected for the calculation of GDD:

1 $$\sum \frac{Daily\;High\;Temperature- Daily\;Low\;Temperature}{2}-Base\;Temperature$$

Climate data (historical GDD and annual precipitation) used in this study were extracted from the Climate Atlas of Canada (2019). The Climate Atlas of Canada (2019) is an extensive collection of historical climate variables (11 variables for hot weather, 9 variables for cold weather, 3 variables for temperature, 7 variables for precipitation, and 8 variables for agriculture) and their associated future projections under two climate change scenarios, covering all of Canada. The interactive map allows users to select areas of interest and explore their climate data.

Annual precipitation (mm) and GDD (base of 4℃; °C·Day) were extracted from 10-km^2^ map cells from the Climate Atlas of Canada for the general areas of the three lakes, for the year of each otolith radius formation (Climate Atlas of Canada 2019). Historical climate data (1950–2013) used in the atlas is provided by Natural Resources Canada (McKenney et al. 2011).

The surface area (km^2^) of the three lakes were estimated using the polygon measurement tool in ArcGIS Online (ESRI 2023). This tool allows the user to trace the perimeter of an object in satellite images to acquire its area. We used surface area as a general measurement of lake depth, as Nõges (2009) found that lakes with larger surface areas tend to be deeper.

### Statistical analyses

Because of the nature of back-calculated datasets, the number of observations declines with increasing age. As such, only ages for which there are at least 25 observations per location were analyzed. This resulted in the exclusion of ages 11–20. The analyses to follow examine ages 1–10 only. In addition, measurements of annuli formed during the year of capture were excluded from analysis, as they are considered incomplete when sampling occurred during the summer. The resulting dataset includes 3783 annulus measurements from 542 individuals.

#### Trend analysis

Trends in mean otolith measurements-at-age for ages 1–10 were detected using two methods with the notrend_test() function from the ‘funtimes’ package in R: a sieve-bootstrap student’s t-test for linear trends and a sieve-bootstrap Mann–Kendall test for monotonic trends (Lyubchich et al. 2023). The purpose of this analysis is to establish whether mean otolith measurements have increased significantly between 1984 and 2013 and to determine which age classes are affected.

#### Model development

All statistical analyses were done using R Statistical Software (v4.3.1; R Core Team, 2023). The data were compiled into a single dataset, which was then split into a training data set (70%) for model development, and a test data set (30%) to cross-validate the model. The training dataset was used to develop the growth models, which were then applied to the test dataset to rule out overfitting and ensure model robustness. A series of model validation plots (distribution of residuals, variance of residuals, and residuals plotted against covariates) were produced to confirm the model assumptions were met. The simulateResiduals() function from ‘DHARMa’ was also applied to the models to detect potential issues in dispersion and heteroskedasticity (Hartig 2024). Data exploration was conducted on the entire dataset following the protocol described by Zuur et al. (2010).

Annulus lengths for age-1 were not taken due to difficulty determining the boundary between the nucleus and the first annulus. As such, only radial measurements are taken for age-1 (i.e., from the centre of the nucleus to the end of age-1 growth). Since younger age classes are thought to be disproportionately affected by climate change due to their shallow habitats, age-1 radial lengths were modelled separately to not overlook such a vulnerable age class. The development of both models was done following the protocol described in Zuur and Ieno (2016). This protocol involves determining the dependency structure of the data, presenting and fitting the model, model validation, interpretation of numerical outputs, and visualizing the model (Zuur and Ieno 2016).

#### Annulus length model

A mixed effect model was used to account for the repeated measurements of each individual, individual variation in growth, temporal autocorrelation, and similarities in growth during the same year. Sample ID and year was included as random intercepts to account for this dependency structure. An overparameterized model was fitted using the ‘mgcv’ package (Wood 2011). A reduced model was reached by backward stepwise selection where predictors are sequentially dropped and compared to the full model by likelihood ratio tests (LRT) using the ‘mgcv’ package (Wood 2011). Terms were removed from the final model when LRT indicated no improvement in fit with the additional parameter.

#### Age-1 radius model

A random intercept of year was necessary for modelling age-1 radius length to account for cohort effects. As such an overparameterized mixed effect model was fitted using the ‘glmmTMB’ package (Brooks et al. 2017). Backward stepwise selection was used to reduce the model using LRT in base R, dropping terms that did not significantly improve the model fit (R Core Team 2023).

## Results

### Trend analysis

**Fig. 2:**
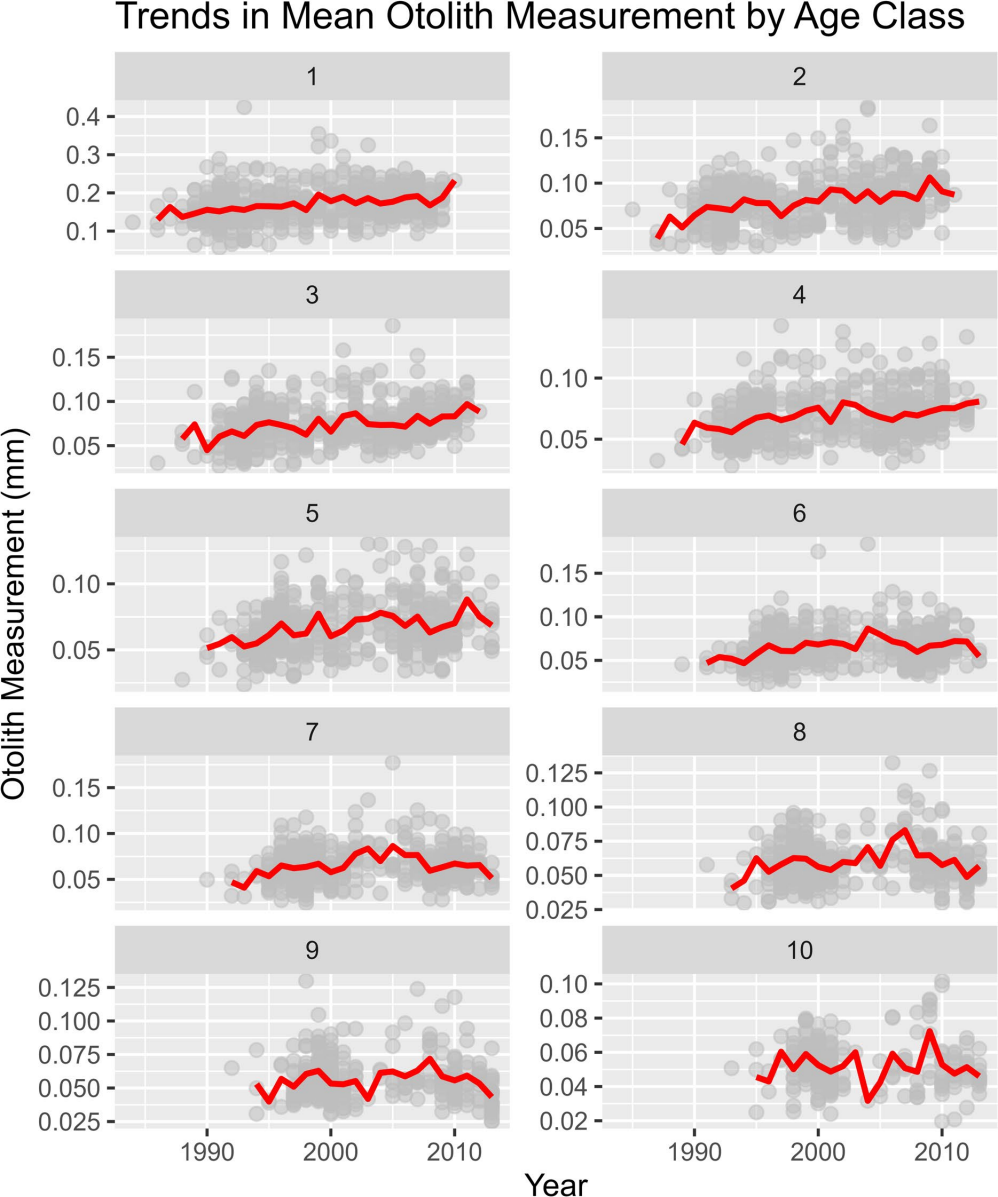
Mean otolith growth-at-age over time showing the observed increase in mean otolith measurements across age classes. The grey points represent observed otolith measurements. The red lines represent the mean otolith measurement over time. Significant trends were detected in ages 1–6 only. Note that for age-1, radius length was used while the remaining age classes are investigated using annulus lengths

Of the 10 age classes, only ages 1–6 and 8 were found to have significant trends in mean otolith measurements over time (Fig. 2). The trends in ages 1–6 were all found to be linear. Ages 1–3, 5, and 8 were also found to have significant monotonic trends in mean otolith measurement. No trends were detected in ages 7 and 9–10. Overall, it appears that the magnitude of the positive trend decreases with age.

### Annulus length model

A Cleveland dotplot revealed a single outlier with an annulus length of 0. It was removed, as it was determined to be a measurement error. A histogram of annulus lengths indicated they have a right-skewed distribution, suggesting a gamma distribution may better describe the positive non-zero data, compared to a Gaussian distribution. The relationships between the response and predictors were visually assessed using ‘ggplot’ scatterplots with gam-smoothed curves (Wickham 2016). Non-linearity was found in GDD, annual precipitation, year, and age, leading to the choice of a generalized additive mixed model (GAMM).

Three interactions were found using coplots in base R (R Core Team 2023). Larger annulus lengths were associated with high growing degree days in young ages (< 5 years). Large annulus lengths were also associated with less annual precipitation in ages 2 and 3. Finally, age-specific annulus lengths differed between stocks. Variance inflation factors (VIF) revealed location and surface area are aliased, resulting in surface area being dropped from the model. All other covariates have VIF values below 2.

From backward stepwise selection, only sex was dropped from the model, as it did not improve the model fit. The interaction between age and location was also found to have an insignificant contribution to fit, however, it was retained as it allows for variation we would expect to see between younger age classes across locations. Thus, the reduced model is as follows:

2 $$\begin{aligned}{Annulus\;Length}_{ijk}\sim Gamma(\mu_{ijk})log(\mu_{ijk})&=s\left({Annual\;Precipitation}_{ijk}\right)+s\left({GDD}_{ijk}\right)+s\left({Age}_{ijk}\right)\\&+{Location}_{ijk}+ti\left({GDD}_{ijk}\times{Age}_{ijk}\right)+ti\left({Annual\;Precipitation}_{ijk}\times{Age}_{ijk}\right)\\&+s({Age}_{ijk}\times{Location}_{ijk})+{Sample\;ID}_j+{Year}_k\end{aligned}$$

where *Annulus Length*_ijk_ (continuous; mm) is the *i*^th^ otolith annulus length from *Sample ID*_j_ in *Year*_k_. Three thin-plate splines were included in the model; s(*Annual precipitation*_ijk_) (continuous; mm), is the annual precipitation of the *i*^th^ annulus length from *Sample ID*_j_ in *Year*_k_, s(*GDD*_ijk_) (continuous; °C*Day) is the corresponding growing degree days with a base of 4 °C and s(*Age*_ijk_) (continuous; years) is the corresponding age of the individual at annulus measurement in *Year*_k_. *Location*_ijk_ (3 level factor; Kipisa, Iqaluit, or Qasigiat) is the presumed natal lake of the *i*^th^ annulus length from *Sample ID*_j_ in *Year*_k_. The tensor product interactions, ti(*GDD*_ijk_* x Age*_ijk,_) and ti(*Annual Precipitation*_ijk_* x Age*_ijk_), represent the disproportionate effects of GDD and Annual Precipitation on younger ages. The smoothed interaction, s(*Age*_ijk_* x Location*_ijk_), allows for variation in annulus lengths-at-age between locations. *Sample ID*_j_ and *Year*_k_ are the random intercepts, assumed to follow normal distributions with mean 0 and variance ${\sigma }^{2}$.

No significant issues were detected during model validation, allowing for interpretation of the effects of each variable on otolith annulus length. All variables were found to be significant at the 5% level (*p* < 0.05; Table 1), except for Location Kipisa. Of the three locations, Qasigiat had the largest annulus lengths. The estimated degrees of freedom (EDF) in Table 1 describe the non-linearity of the smooth terms and interactions. When EDF is equal to 1, the term is linear. The larger the EDF, the more wiggly the term. 20.4% of the deviance was explained by the model.

**Table 1:** Table of the Model 1 summary statistics

Parametric coefficients				
	Estimate	Standard error	*t*-value	*p*-value
(Intercept)	− 2.710	0.008	− 320.28	< 0.001
Location Kipisa	− 0.014	0.012	− 1.19	0.24
Location Qasigiat	0.050	0.014	3.70	< 0.001
Approximate significance of smooth terms				
	Edf	Reference df	*F*	*p*-value
S(precipitation)	4.776	5.810	7.02	< 0.001
S(GDD)	6.074	7.175	6.81	< 0.001
S(age)	3.722	4.536	34.51	< 0.001
ti(GDD, age)	6.793	8.341	4.95	< 0.001
s(age): Iqaluit	0.003	0.005	0.004	0.99
s(age): Kipisa	3.785	4.612	6.58	< 0.001
s(age): Qasigiat	1.006	1.012	6.75	0.01
ti(precipication, age)	8.591	11.020	3.27	< 0.001

**Fig. 3:**
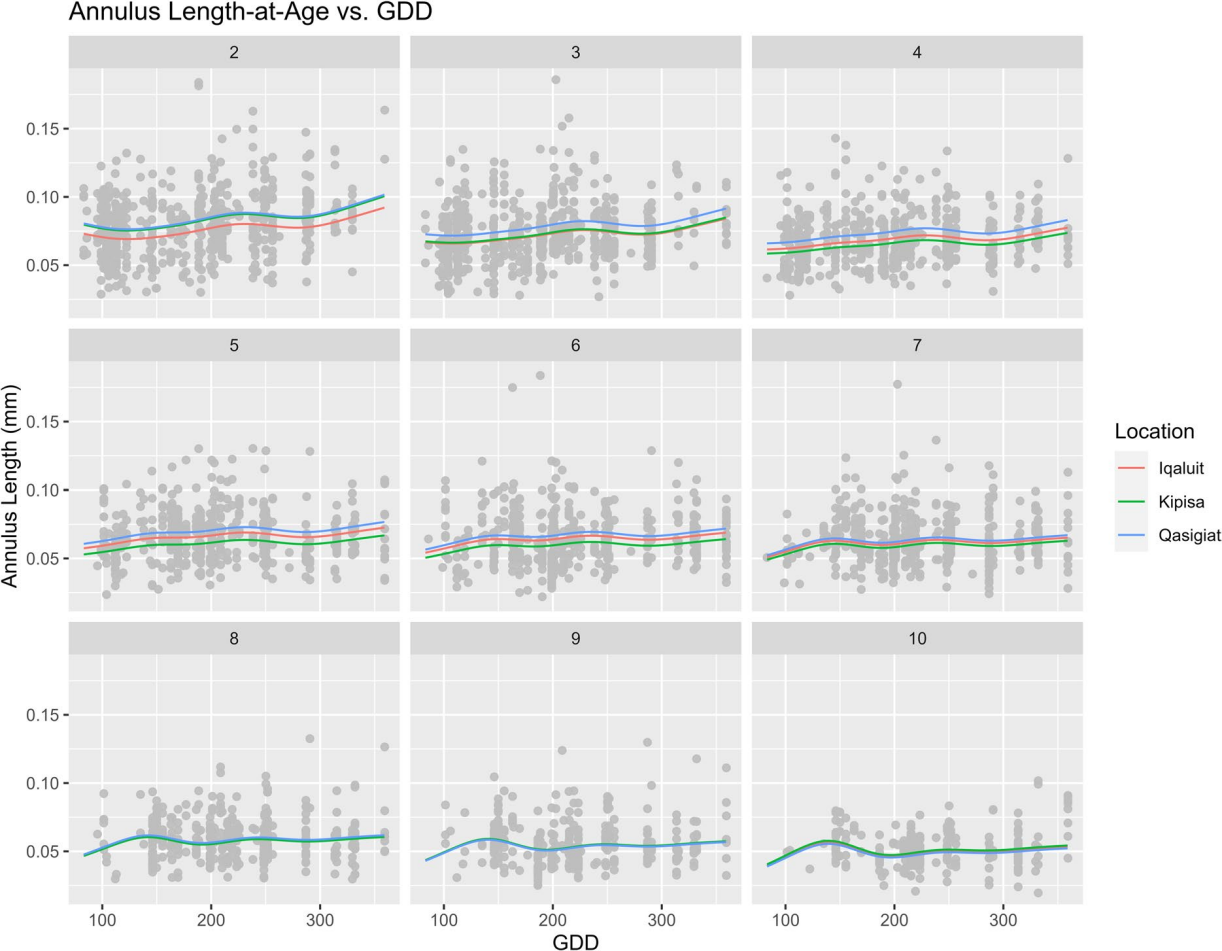
A series of plots demonstrating the effect of GDD on annulus length across ages 2–10. Predicted annulus length is represented by a red line for Iqaluit, green for Kipisa, and blue for Qasigiat. The grey points are the observed annulus lengths across all locations

To visualize the effects of GDD and annual precipitation independently, we created plots as follows: a new dataset was made where GDD and Age varied from the observed minimums to maximums while holding precipitation constant at its mean observed value (304.88 mm). This new dataset was then applied to model 1 using the ‘mgcv’ package to predict annulus lengths and standard errors for each age across the range of GDD values (Wood 2011). Results were then plotted in Fig. 3. GDD was found to have an overall positive effect on annulus length across all ages.

**Fig. 4:**
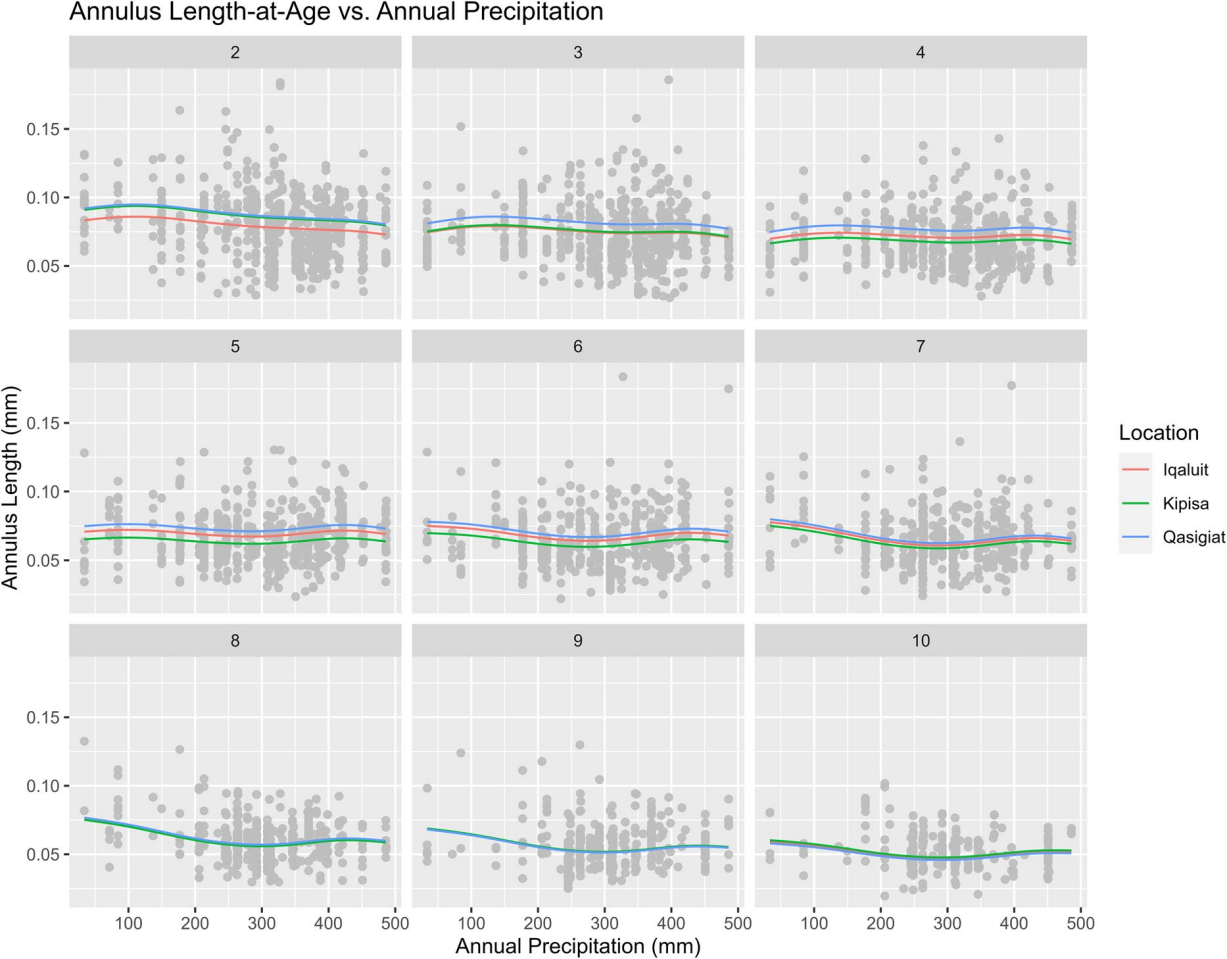
A series of plots demonstrating the effect of annual precipitation on annulus length across ages 2–10. Predicted annulus length is represented by a red line for Iqaluit, green for Kipisa, and blue for Qasigiat. The grey points are the observed annulus lengths across all locations

The same method was used to isolate the effects of annual precipitation, by holding GDD constant at its mean value (210.59 °C*Day). Figure 4 indicates precipitation had a negative effect on annulus length for age classes 2 and 7–10. The most pronounced negative effect of precipitation was seen at age-2. For ages 3–6, annual precipitation seems to have had a marginal effect on annulus length.

### Age-1 radius length model

No outliers were found within the age-1 radius lengths. The radius lengths were found to be normally distributed, suggesting a typical Gaussian distribution should fit the data well. Relationships between the response and each covariate were found to be linear. Year was included as a random intercept to account for variation between cohorts, resulting in the use of a linear mixed model. No biologically relevant interactions were found. Backward stepwise selection indicated none of the variables should be dropped from the model. The following model was produced based on data exploration to describe age-1 radius length:

3 $${Age1\;Radius}_{ij}\sim N(\mu_{ij},\sigma){Age1\;Radius}_{ij}\sim{GDD}_{ij}+{Annual\;Precipitation}_{ij}+{Location}_{ij}+{Year}_j$$

where *Age1 Radius*_ij_ (continuous; mm) is the age-1 radius length for the *i*^th^ observation of *Year*_j_, *GDD*_ij_ (continuous; °C*Day), *Annual Precipitation*_ij_ (continuous; mm) and *Location*_ij_ (3 level factor) are the corresponding GDD, annual precipitation, and location of the *i*^th^ observation of *Year*_j_. *Year*_j_ is the random intercept, assumed to be normally distributed with mean 0 and variance ${\sigma }^{2}$.

**Fig. 5:**
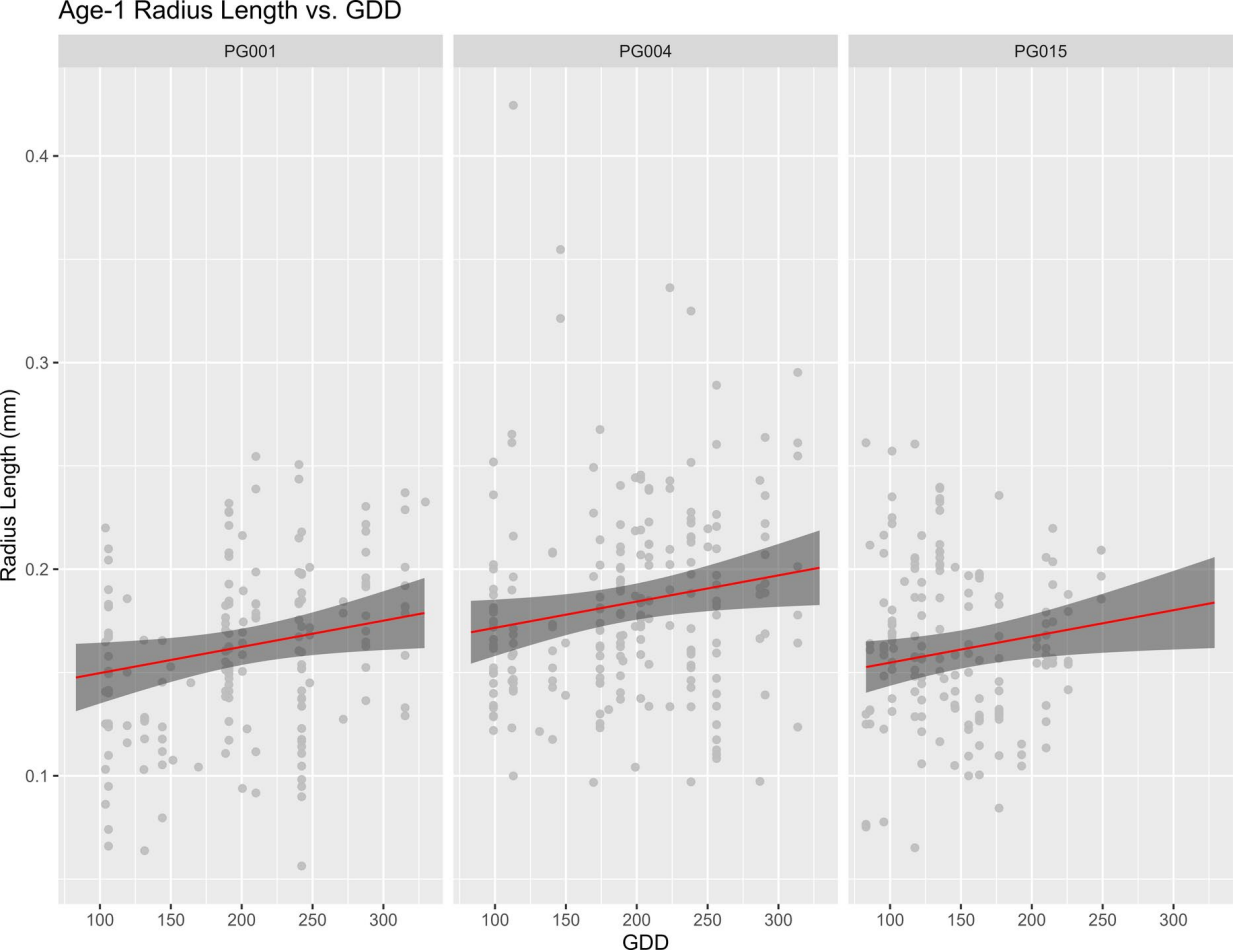
Plot showing the effect of GDD and location on age-1 radius length. The grey shadow represents the 95% confidence interval around the predicted age-1 radius length, represented by the red line. The grey points are the observed age-1 radius lengths

**Fig. 6:**
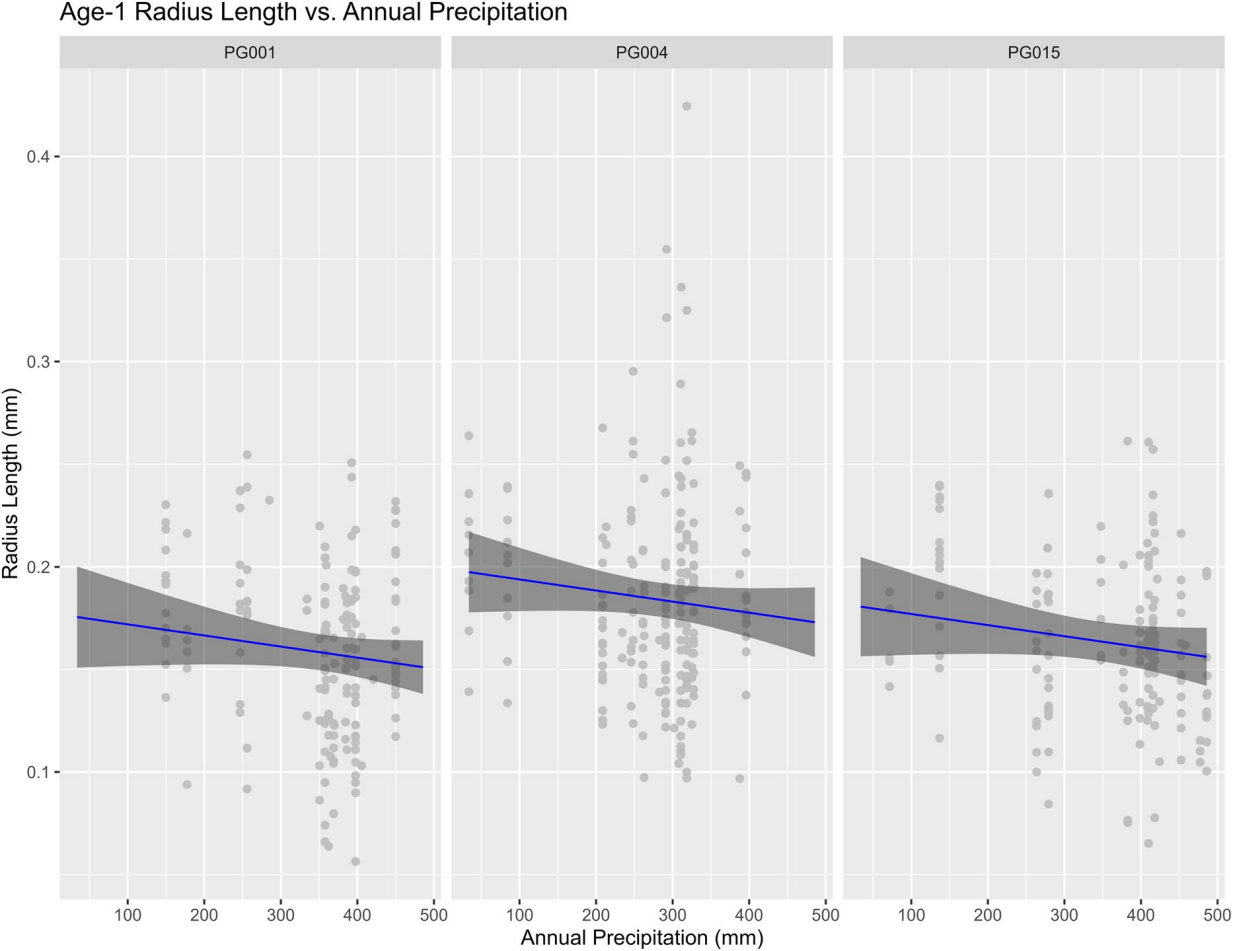
Plot showing the effect of annual precipitation and location on age-1 radius length. The grey shadow represents the 95% confidence interval around the predicted age-1 radius length, represented by the blue line. The grey points are the observed age-1 radius lengths

No significant issues were detected during the model validation or cross-validation process. GDD and Location Kipisa were the only variables found to be statistically significant (*p* < 0.05; Table 2). Similar to annulus lengths for ages 2–10, GDD had a positive influence on age-1 radial length, while annual precipitation showed a small negative influence (Figs. 5 and 6). Age-1 radial lengths in Kipisa were found to be larger than the other two locations. Nakagawa’s R^2^ was calculated using the ‘performance’ package and found to be 0.210 for the conditional model and 0.125 for the marginal (Lüdecke et al. 2021).

**Table 2:** Table of the Model 2 summary statistics

Summary Statistics				
	Estimate	Std. error	*z*-value	*p*-value
Intercept	0.1543	0.020	7.67	< 0.001
GDD	0.0001	0.00006	2.16	0.03
Location Kipisa	0.022	0.005	4.35	< 0.001
Location Qasigiat	0.005	0.006	0.86	0.38
Annual Precipitation	− 0.00005	0.00004	− 1.47	0.14

## Discussion

The main goal of this study was to determine whether the growth of Arctic charr has changed in response to climate change between 1984 and 2013. As anticipated, we found that otolith radius and annulus lengths (and, by inference, charr growth) have increased significantly across age classes (1–10) in concert with increases in temperature (i.e., GDD). However, annual precipitation had an overall negative effect in ages 1–2 and 6–10, and virtually no effect on ages 3–5. Trend analyses indicate that significant increases in otolith measurements have occurred in ages 1–6 and 8 during the time period.

Our finding of increased growth with GDD is consistent with the results of similar studies of charr in Norway and Greenland (Kristensen et al. 2006; Kotowych et al. 2023; Svenning et al. 2024). Svenning et al. (2024) found that young-of-the-year charr growth in Svalbard was positively correlated with summer air temperature. This is consistent with our finding of a positive correlation between GDD, and annulus length (ages 2–10) and age-1 radius, respectively, as winter temperatures below the base temperature of 4 °C would not contribute to cumulative GDD and, therefore, growth. Kotowych et al. (2023) also reported juvenile Arctic charr (ages 1–4) growth increased by 6.8–12.6% with water temperatures (modelled from air temperature) over a similar time period (1986–2016).

As previously mentioned, the growth of charr is expected to increase with temperature up to an optimum (10.3–16.3 °C; Lyytikäinen et al. 2002; Larsson and Berglund 2005a; Beuvard et al. 2022). However, growth is only enhanced by temperatures if food availability is not limited. When food availability is insufficient, metabolic needs cannot be met while maintaining growth at high temperatures, leading to diversions of energy from growth (and possibly reproduction) to support metabolism (Gilbert et al. 2020). Kristensen et al. (2006) studied the effects of temperature increases on growth of landlocked charr. Despite similar warming trends, one population showed increased growth, while in the other, growth was suppressed (Kristensen et al. 2006). The latter habitat was described as poor with limited food availability, demonstrating energetic diversions from growth towards metabolic needs when temperatures rise and energy intake is insufficient (Kristensen et al. 2006). Our current findings did not indicate such trade-offs, suggesting the study lakes are sufficiently productive to support enhanced growth prior to first migration.

Precipitation has been found to have varying effects on the growth of charr, depending on the season and type of precipitation. In the current study, we found annual precipitation had an overall negative influence on growth in ages 1–2 and 6–10. A similar result was found by Kristensen et al. (2006) in a landlocked population of charr, where mean annual precipitation had a negative effect on growth. Svenning et al. (2024) found that greater snow depth (i.e., higher winter precipitation) had a negative influence on growth due to its insulative properties, delaying ice melt. However, Chavarie et al. (2019) reported increased growth in older age classes with spring precipitation only, likely resulting from related increases in estuarine productivity and thus, feeding opportunities. Because annual precipitation was used, it is difficult to infer whether the relationships seen here are due to the influence of rain or snow.

However, greater snowfall could very well be an explanation for the more pronounced effects on growth in ages 1–2 and 6–10. For instance, a large proportion of growth occurs at the commencement of anadromy at ~ 6 years of age for nearby stocks (Loewen 2016; Young et al. 2021). If the snow cover is deep, the ice is insulated and can persist longer. This could then result in delayed migration, possibly explaining the greater decline in growth with precipitation for ages 6–10. While Hammer et al. (2022) found that Arctic charr migration occurred just before the ice breaks up, delayed ice melt could still delay entry into the marine environment. Age-1 and −2 growth show similar trends, which again may be related to snow accumulation, which shortens the growing season (Svenning et al. 2024). For ages 3–5, annual precipitation seems to have marginal effects on growth, perhaps reflecting their movements within the lake prior to first migration. Dubos et al. (2022) found that juveniles greater than 1 year of age had no habitat preference within the freshwater environment, and they were much more mobile than their younger conspecifics. In addition, the risk of predation generally decreases with size, so smaller individuals (thus, younger) may restrict habitat use to shallow areas (Byström et al. 2004). It is expected that the continued decline in snowfall into the future will lead to earlier ice melt (AMAP 2021), thus supporting greater growth.

The models reveal variations in growth between locations in ages 1–6. In addition, the trend analyses indicate significant increases in growth solely in these age classes. As previously mentioned, the mean age at first migration is ~ 6 years in Cumberland Sound (Loewen 2016; Young et al. 2021). The variation seen in growth between locations is likely a reflection of the habitat variation and food availability of the lakes, as older age classes do not appear to have this same variation. For ages 2–6, charr in Qasigiat appear to have greater growth than those in the other two locations. Qasigiat is somewhat unique in comparison to Iqaluit and Kipisa, as it is often inundated with salt water due to its proximity to the marine environment. It has a limited littoral habitat due to its steep edges, reaching depths greater than 21 m, and it is thought that rearing occurs in shallow off-shoot ponds that surround the lake (Loewen 2008). Martin and Tallman (2013) reported that charr in this system appears to reach an asymptotic length of ~ 600 mm around 10 years of age. Other stocks in Cumberland Sound do not appear to share this characteristic (Harris and Tallman 2010; Martin and Tallman 2013). One possible explanation for the greater observed growth in Qasigiat is that the inflow of marine water may carry nutrients that could support greater juvenile growth. Alternatively, Gilbert et al. (2022) found that Arctic charr behaviourally thermoregulate by moving to cooler thermal zones in a body of water to support metabolic processes. We know Qasigiat is deep (> 21 m), suggesting it may have more extensive thermal stratification than the two other lakes. As such, Qasigiat charr may be able to maximize growth by moving to different thermal zones within the water column.

Kipisa was found to have greater age-1 radial lengths than Iqaluit and Qasigiat. These measurements not only include age-1 growth but also larval growth as it includes the nucleus. This stock is quite distinguishable as Kipisa charr tend to have deeper bodies, smaller heads, and lighter body colour in comparison to other Cumberland Sound stocks (DFO 2005). Studies have found that larger females are able to produce larger eggs with greater yolk sac content than smaller females (Eiríksson et al. 1999; Leblanc et al. 2016). Larger eggs result in larger embryos with greater yolk reserves. As such, the larger age-1 radius in Kipisa may be attributed to larger embryos due to a maternal effect. However, egg size has also been attributed to spawning habitat. Beck (2019) found that anadromous charr spawning in rivers produced smaller eggs than those that spawned in lakes. Takatsu et al. (2023) also found this to be true with charr in Greenland, although not statistically significant. While it is hypothesized that Kipisa charr spawns in the river above the lake, the spawning sites of most stocks in the area are not well studied.

Growth of ages 7–10 does not appear to vary between stocks. This pattern is likely linked to both a reduced growth rate and abundant and likely similar resource availability in the marine environment. Between the ages of 8 and 13, Arctic charr in these stocks begin to spawn (DFO 2005; Martin and Tallman 2013; Martin et al. 2023). As a result, energy is likely to be diverted from somatic growth towards reproduction, depressing the growth rate. In addition, this age range is also exploiting the rich marine environment. All three of the stocks studied here migrate to Cumberland Sound, where we can presume resource availability and environmental conditions are more similar than those of the freshwater lakes. In addition to food availability, we know the temperature of the marine environment remains fairly stable and not as influenced by atmospheric temperature as smaller bodies of water. Each of these aspects would contribute to more homogeneity of growth between stocks.

Previous assessments (DFO 2005, 2013, 2023) on these stocks have not detected any significant changes to growth during this time period. Our current findings underline the importance of conducting long-term growth studies, as shorter time scales used for DFO stock assessments (5 years) are likely too short to detect such changes. Seeing as charr are relied on for both subsistence and commercial fishing in northern communities (Babaluk et al. 2010; Roux et al. 2011), it is important to consider the broader implications that Arctic warming may have on stock biomass. In the short term, climate change will likely have positive effects on both commercial and subsistence harvests. As we found here, age-specific growth has already increased with GDD between 1989 and 2013. In addition, Grenier and Tallman (2021) found that juvenile charr with faster growth rates tend to become anadromous, suggesting a possible increase in the preferred anadromous ecotypes with further warming. Spawning typically does not occur on an annual basis due to the short growing seasons with low temperatures and limited food availability (Johnson 1980; Dutil 1986). Females especially, may require two or more years to accumulate enough energy stores for egg development (Dutil 1986; Parker and Johnson 1991). However, with warmer temperatures comes a longer ice-off season, possibly prolonging the marine feeding period. As such, fecundity may increase by allowing for more frequent spawning. Each of these aspects could lead to greater stock biomass, allowing for larger quotas assuming body condition remains good.

However, long-term effects of the warming climate will be negative and likely result in a lower biomass. Not only is growth rate expected to decline, but mortality is likely to increase. In migrating charr, temperatures above 16 °C were found to impair recovery from exhaustive exercise and at 21 °C resulted in arrhythmia (Gilbert et al. 2020). It is also expected that species like brook charr, *Salvelinus fontinalis* (Mitchell 1814), may move northward, outcompeting Arctic charr (Bommersbach et al. 2024). With that said, it is embryos and juveniles that are likely to experience the most immediate consequences due to their low tolerance for warm temperatures (Elliott and Elliott 2010). Indeed, no trends in growth after the average age of first migration were found in the present study likely due to the shallow nature and disproportionate warming of juvenile habitats. Lakes with limited juvenile habitat like Qasigiat may be more vulnerable to higher mortality in eggs and juveniles as temperatures continue to increase. Since small individuals select habitats based on the risk of predation and food availability (Damsgård and Ugedal 1997; Byström et al. 2004), rising temperatures may force juveniles to occupy higher risk environments to escape the heat. Eventually, the conditions of these freshwater lakes may deteriorate to the point of becoming uninhabitable.

## Conclusion

We have found that Arctic charr in Cumberland Sound has already experienced changes in growth since at least 1984, in concert with climate change-associated increases in GDD and annual precipitation. Qasigiat charr were found to have significantly larger annulus lengths in ages 2–6 in comparison to Iqaluit and Kipisa. These differences are thought to be attributed to differences in lake habitats and presumed extensive thermal stratification resulting in maximized growth. In contrast, Kipisa was found to have greater age-1 radial lengths than the other stocks, hypothesized to be related to a maternal effect, thus producing larger embryos. GDD was found to have a positive influence on growth across ages, while annual precipitation had a negative effect in ages 1–2 and 7–10. These patterns are thought to be tied to the shallow nature of habitats occupied by younger ages and impediments to migration for older ages. The results of the models and trend analyses point to a disproportionate effect of climate change on individuals before their first migration. With that said, the stock assessments conducted on the samples used in the current study failed to detect any significant trends in growth, demonstrating the need for more thorough growth studies to aide in the management of subsistence and commercial fisheries in a warming Arctic.

## Data Availability

Data will be made available through the Open Government Portal (https://open.canada.ca/en) within 2 years of the completion of the project.
